# Construction of the waaF Subunit and DNA Vaccine Against *Escherichia coli* in Cow Mastitis and Preliminary Study on Their Immunogenicity

**DOI:** 10.3389/fvets.2022.877685

**Published:** 2022-05-12

**Authors:** Hua Wang, Ligang Yuan, Tao Wang, Lu Cao, Fukang Liu, Juanjuan Song, Yong Zhang

**Affiliations:** Gansu Key Laboratory of Animal Generational Physiology and Reproductive Regulation, College of Veterinary Medicine, Gansu Agricultural University, Lanzhou, China

**Keywords:** *Escherichia coli*, waaF, pcDNA3.1-waaF, waaF recombined protein, immunogenicity

## Abstract

*Escherichia coli* (*E. coli*) is one of the major pathogenic bacteria in bovine mastitis, which usually triggers systemic symptoms by releasing lipopolysaccharide (LPS). waaF is the core in LPS pathogenicity. In this study, a new waaF vaccine candidate was identified, constructed with the pcDNA3.1 (+)HisB-waaF plasmid to create to a DNA vaccine (pcwaaF), and transfected into MCF-7 cells to produce recombinant waaF subunit vaccine (rwaaF). After that, the safety of the two vaccine candidates was evaluated in mouse model. Immunogenicity and mortality of challenged mice were compared in 20 and 40 μg per dose, respectively. The results showed that rwaaF and pcwaaF were successfully constructed and the complete blood count and serum biochemical indicated that both of the vaccine candidates were safe (*p* > 0.05). In addition, histopathological staining showed no obvious pathological changes. The immune response induced by rwaaF was significantly higher than that of pcwaaF (*p* < 0.01), indicated by levels of serum concentration of IgG IL-2, IL-4, and IFN-γ, and feces concentration of sIgA. Survival rates of mice in rwaaF groups (both 80%) were also higher than in the pcwaaF groups (40 and 50%, respectively). Comparing the safety, immunogenicity, and *E. coli* challenge of two vaccine candidates, rwaaF had the better effect and 20 μg rwaaF was more economical. In conclusion, this study demonstrates the utility of a new *E. coli* vaccine and provides a rationale for further investigation of bovine mastitis therapy and management.

## Introduction

Coliform mastitis is one of the most common diseases in the dairy industry worldwide. It can cause severe acute inflammation with toxemia, high fever, and decreased milk production (1, 2) and lead to serious economic losses (3, 4). In addition, the incidence of coliform mastitis has increased in the past few decades (5). It is among the environmental pathogens that occur wherever there are cows, such as the bovine digestive system (5). Therefore, ruminant feces are an important infectious source of *Escherichia coli* (*E. coli*) that can disseminate directly into the surrounding environment (5).

The virulence factors of the pathogenic *E. coli* include lipopolysaccharide (LPS) (6) and flagella (7) among others. LPS is the key to the cause of mastitis. When *E. coli* infects and proliferates in the mammary duct system, it releases LPS that causes breast infection (8) and activates the host's immune system (9). Although LPS can be engulfed and destroyed by immune cells, a large amount of LPS is released and causes systemic symptoms in the host.

LPS comprises the outer cell-wall constituents of Gram-negative bacteria. It is composed of three regions: an O-antigen of repeating oligosaccharide units, lipid A, and a core oligosaccharide. Among them, bacteria can be classified into smooth (S-) and rough (R-) types according to the presence of O-antigen (10). Lipid A is the most conserved part of LPS and is connected by lipid glucosamine disaccharide and phosphate through pyrophosphate bonds (11). The core oligosaccharide is separated into an outer and inner core; the outer core has a different composition and configuration in diverse strains, whereas the inner core is composed of heptose residues and 2-keto-3-deoxyoctanoic acid, which is highly conserved and is a common structure in all strains of *E. coli* (12). With the catalysis of heptosyltransferse (waa), lipid A is linked to the Kdo disaccharide of oligosaccharide forming the Kdo2-lipid A group, which is the most conserved in LPS and is called a toxic center (12). waaF is a heptosyltransferse II gene, which is the second Hep to Kdo2-lipid A (13). The loss of waaF results in a severely truncated LOS structure (14). Compared with other genes in waa family, the waaF mutant showed stronger hydrophobicity, autoaggregation, and outer membrane permeability. Furthermore, the effect on the flagella assembly, chemotaxis, and pro-inflammatory responses of bacteria is more significant (13, 15). Therefore, waaF is not only related to the adhesion of pathogenic bacteria, but also involved in the host-pathogen interaction. Therefore, waaF is an ideal gene as a target antigen for a vaccine feasibility study.

Among newly developed vaccines, DNA and subunit vaccines have been a focus of research. In DNA vaccines, a recombinant eukaryotic expression vector encoding a certain protein antigen is directly injected and expressed into the animal, and the resulting antigen activates the immune system, thus inducing specific humoral and cellular immune responses (16); the subunit vaccine is a recombinant protein vaccine that combines the main protective immunogen of pathogenic bacteria. Both of them not only have the advantages of being simple, effective, and more targeted, but also of being effective against a wide variety of pathogens simultaneously, or against a single pathogen by multilocus antigen (17). Many DNA and subunit vaccines have been developed against *E. coli* mastitis in dairy cows (18), but there are no reports on a waaF vaccine.

In this study, waaF of *E. coli* that was separated from milk was cloned and expressed in eukaryotic vector pcDNA3.1 (+)-HisB to construct the pcDNA-waaF vaccine (pcwaaF). Afterwards, it was transfected into MCF-7 cells to purify the waaF recombinant protein (rwaaF). Furthermore, we compared the safety, immunogenicity, and immune protection of waaF in two different vaccines that were used in a murine model.

## Materials and Methods

### Animals and Institutional Approval

We purchased 110 lactating BALB/c female mice (6–8 weeks old) from the Institute of Veterinary Medicine, Chinese Academy of Agricultural Sciences (Lanzhou, China). This study was approved by the Academic Committee of Gansu Agricultural University and the National Natural Foundation of China (Grant No. 202009264), and researchers followed the guidelines for the protection and use of experimental animals of the Institute of Experimental Animal Resources of the National Research Council.

### Methods

All molecular tests are performed according to the Molecular Cloning: A Laboratory manual (Fourth Edition) (19) specifications and Vaccine Technology (20).

#### Construction of PcDNA3.1–waaF (pcwaaF) and waaF Recombinant Protein (rwaaF)

##### Cloning of the waaF Gene

The waaF primers (Shanghai Bioengineering Co., Shanghai, China) (waaF-F-*KpnI*, 5′TGGGTACCAAGATGGGATGAAAATAC3′; waaF-R-*BamHI*; 5′CACACTGGATCCTCAGGCTTCC3′, the restriction sites are underlined) were added to *E. coli* NC-00913.3 (3794929-3795975). The standard PCR (BIO-RAD, USA) conditions were as follows: initial denaturation at 95°C for 5 min, followed by 35 cycles of 94°C for 60 s and annealing at 60°C for 45 s and then 72°C for 60 s, and final extension for 10 min at 72°C. PCR products were resolved by 1.0% agarose gel containing 0.1 μg/ml Golden View^TM^ and recovered with a Star Prep Gel Extraction Kit (TransGen Biotech Co., Beijing, China). Part of an amplified waaF gene was sent to Shanghai Bioengineering Co., Ltd for sequencing and the remainder stored at −20°C.

The PCR products of waaF was cloned into the *KpnI*–*BamHI* site of the pGEM-T-easy vector (Promega, USA) after digestion by two restriction endonucleases (TransGen Biotech Co., Beijing, China), transformed into *E. coli* DH5α (TransGen Biotech Co., Beijing, China), and then proliferated in Luria-Bertani (LB; Solarbio, Beijing, China) broth agar medium containing X-gal 40 μl (20 mg/ml) and IPTG 4 μl (200 mg/ml), (Solarbio, Beijing, China) for 4 h. The white plaque was removed and cultured in LB broth with anti-ampicillin for 8 h. Then extracted plasmid DNA was extracted and sent to Shanghai Bioengineering Co., Ltd. for sequencing.

##### Construction of pcwaaF

pcDNA3.1 V5-HisB (pc DNA 3.1), (Invitrogen, Shanghai, China), and the positive plasmid was split with *KpnI* and *BamHI* (Promega, USA) at 37°C and ligated at 16°C for 12 h by T4 DNA ligase (Promega, USA).

The resultant ligated pcDNA3.1-waaF (pcwaaF) was cloned into *E. coli* DH5α (For details, see Section Cloning of the waaF Gene). The positive plasmid DNA was extracted and purified by the minibest DNA purification Kit (TaKaRa, Dalian, China), digested with *Kpn*I and *BamHI*, and sent to Shanghai Bioengineering Co., Ltd for sequencing again. The purity was measured by spectrophotometry using the A260/280 absorbances (Quawell, America).

##### Construction of rwaaF

*Transfection.* The pcwaaF plasmid DNA was extracted and quantitated as 1 μg/μl, and then digested with *KpnI* and *BamHI* and linearized. MCF-7 cells (Institute of Bioengineering, Fudan University, Shanghai, China) were grown on Dulbecco's modified Eagle's medium supplemented with 10 % fetal bovine serum (Sigma, Australia). Every 2 days, 2.5 ml fresh medium was added to the culture. Cells (1 × 10^5^) were seeded in a 24 well plate (TransGen Biotech Co., Beijing, China) and transfected 24 h later. Furthermore, 90% of the wells were filled with cells and the medium used in the step was without any antibody. Lipofectamine 2000 (TransGen Biotech Co., Beijing, China) was used for transfection. The ratio of linearized plasmid DNA (1 μg) and lipofectamine (μl) was 1: 2. After 48 h incubation, the transfection products were selected by medium with G418 in a 96-well plate (TransGen Biotech Co., Beijing, China). The concentrations of G418 were 500, 600, 700, 800, 900, and 1,000 μg/μl. After 15 d, the suspected positive cells were harvested and assayed for the result of transfection.

*Quantitative RT-PCR.* RNA was extracted by Trizol when waaF positive cells were about 90% in six flasks. Then the concentration and OD 260/280 values was analyzed by spectrophotometer and samples diluted to 200 mg/μl. All RNA samples underwent RT-PCR as well as real-time quantitative reverse transcriptase-polymerase chain reaction (qRT-PCR) studies. The concentration of single-strand cDNA (TransGen Biotech Co., Beijing, China) was diluted to 200 mg/μl. RT-PCR analysis was performed on a LightCycler 96® system (Roche, Basle, Switzerland). All reactions were run in triplicate. The cycle threshold (ct) method was used to calculate values and the GAPDH were used to normalize the level of mRNA.

The GAPDH gene (Qinke, Xi'an, China) was designed by Primer 6.0 and the sequences were shown in Table 1. The reaction and the subsequent melting curve protocol were performed in a final volume of qRT-PCR assay with 25 μl, containing 0.5 μl cDNA, 1 μl (10 mM) of each primer, 12.5 μl SYBR premix EX TaqTM II (2 ×) (TransGen Biotech Co., Beijing, China), and 0.5 μl ROX Reference Dye II (50 ×) (TransGen Biotech Co., Beijing, China). Conditions for qRT-PCR included 95°C for 30 s, and 40 cycles at 95°C for 10 s, 60°C for 30 s, and an extension time for the melt curve from 60 to 95°C. The cooling step was performed at 40°C for 10 s (ramp rate of 1.5°C/s).

**Table 1:** Primers for qRT-PCR.

**Gene name**	**Primers sequence**	**Tm (**°**C)**
GAPDH	GGTACCAGGGCTGCTTT	60
	CTGTGCCGTTGAACTTGC	
waaF	GCCTTCCCACGACTGTGTAT	58
	GGAAAAGCTGTTGCCAGAAG	

##### Purification of Recombinant Protein and Western Blot (WB).

The total protein formed by the cultured transfected cells with the highest expression was screened by qRT-PCR and other two vials of positive cells were extracted using a total protein extraction kit (TransGen Biotech Co., Beijing, China) separated by polyacrylamide gel electro phoresis (SDS-PAGE), and the target band cut down and transferred to polyvinylidene fluoride (PVDF, TransGen Biotech Co., Beijing, China) using anti-His Tag monoclonal antibody (AbM59012-18-PU, 1:2,000), (Beijing Protein Innovation, Beijing, China) at 4°C for 12 h. After washing for 3 × 10 min by tris-buffered saline with Tween 20 (TBST) (Solarbio, Beijing, China), PVDF was incubated in Rabbit Anti-Mouse IgG [ab 6728, H&L (HRP)], (Abcam, USA) secondary antibody at 37°C for 2 h, and washed for 1.5 h, as well as exposed by to chemiluminescence detection.

After that, the most highly expressed protein was purified by Ni-NTA affinity chromatography (TransGen Biotech Co., Beijing, China) according to the manufacturer's directions. The purified protein was dialyzed against PBS for 32 h at 4°C and concentrated with renaturation solution (0.1 M Tris, 1 mM glutathione, and urea at concentrations of 1, 2, 4, and 8 M).Protein was determined by HPLC C18 column (Thermo, America); the concentration of the sample was 250 μg/ml, and the volume of loading was 20 μl. Then the protein was quantified by the Protein Quantitative Kit (TransGen Biotech Co., Beijing, China), adjusted to 900 μg/ml, and stored at −80°C.

#### Vaccine Safety Assessments

We randomly divided 50 female lactation BALB/c mice (8–10 weeks old) into five groups with 10 mice in each group. The treatments are shown in Table 2. The pcwaaF or rwaaF was injected subcutaneously, whereas 10 control mice were inoculated with PBS.

**Table 2:** Vaccine safety assessment procedures.

**The vaccine type**	**Inoculum**		
	**0 d**	**7 d**	**14 d**
PBS (μg)	80	80	80
rwaaF (μg)	60	60	60
rwaaF (μg)	80	80	80
pcwaaF (μg)	60	60	60
pcwaaF (μg)	80	80	80

During the immunization period, the energy, appetite, and death of mice were observed and recorded.

At 7 days after the last vaccination, intraperitoneal 1% pentobarbital sodium at a dose of 0.5 mg/10 g was used for anesthesia, and then two tubes of heart blood samples were collected, one with anticoagulant added for routine blood work and the other isolated from the other sample. The following parameters were evaluated: red blood cell count (RBC), hemoglobin concentration (HB), hematocrit (HCT), mean corpuscular volume (MCV), mean corpuscular hemoglobin (MCH), mean corpuscular hemoglobin concentration (MCHC), platelet count (PLT), white blood cell count (WBC), and red blood cell volume distribution width (RDW). Blood biochemical indexes were as follows: alanine transaminase (ALT), aspartate aminotransferase (AST), alkaline phosphoesterase (ALP), total bilirubin (T-BIL), blood urea nitrogen (BUN), creatinine (CR), total protein (TP), albumin (ALB), and glucose (GLU).

The mice were sacrificed by cervical dislocation and the mammary glands, liver, kidney, and spleen were removed and stained to observe the pathological changes.

#### Immunogenicity Analysis

As shown in Table 3, 60 identical mice were randomly divided into six groups with 10 mice in each group and vaccinated three times in total, once a week. The dose and varieties of injections were as follows: rwaaF 20 μg (Goup1, G1), rwaaF 40 μg (Goup2, G2), pcwaaF 20 μg (Goup3, G3), pcwaaF 40 μg (Goup4, G4), PBS 20 μg (Control group 1, C1), and PBS 40 μg (Control group 2, C2).

**Table 3:** Vaccine immunogenicity and *E. coli* challenge.

**The vaccine type**	**Inoculum**			***E. coli* challenge(CFU)**
	**1 d**	**7 d**	**14 d**	
C1	20	20	20	4 ×10^6^
C2	40	40	40	4 ×10^6^
G1	20^a^+20^b^	20^a^+20^d^	20^a^	4 ×10^6^
G2	40^a^+40^b^	40^a^+40^d^	40^a^	4 ×10^6^
G3	20^c^+20^b^	20^c^+20^d^	20^a^	4 ×10^6^
G4	40^c^+40^b^	40^c^+40^d^	40^a^	4 ×10^6^

a *rwaaF*.

b *Complete Freund's adjuvant (CFA)*.

c *pcwaaF*.

d *Incomplete Freund's adjuvant (IFA)*.

*The toxicity or adverse effects of vaccinated rwaaF (60 and 80 μg), pcwaaF (60 and 80 μg), and PBS against BALB/c mice were evaluated by complete blood count and serum biochemical profile. ANOVA test was used for the comparison of blood parameters between vaccinated and non-vaccinated group. P <0.05 were considered significant*.

Over the next 4 weeks, the blood samples were collected from the tail tip and feces of mice every week after immunization and serum IgG (Abcam, USA) and fecal sIgA (Abcam, USA) was determined by ELISA. After the last immunization, heart blood was collected with a disposable needle and IL-2, IL-4, and IFN-γ (Abcam, USA) were detected, determined by ELISA.

#### *E. coli* Challenge in Mice

At 7 days after cardiac blood collection, each mouse was subcutaneously injected with an *E. coli* dose of 4 × 10^6^ CFU and the death rate of mice was recorded.

### Measurement and Statistical Analyses

All assays in this study were performed in three independent biological experiments with at least three replicates.

PCR data was imported by the LightCycler 96® software (Roche, Basle, Switzerland) to perform analysis according to the instructions listed in the system. These data were copied in Office Excel 2007 (Microsoft Corporation, Redmond, WA, USA) and the Efficiency values, *R*^2^, and Cq standard deviations were analyzed.

The sections were photographed by a NIKON ECLIPSE 80i mi croscope camera system and five non-repetitive fields (bar = 20 μm for the mammary gland, liver, and kidney and 10 μm for the spleen) were randomly selected for each section.

The gray curve of the Western blot expression band was analyzed by using Image J 1.48. The area under the peak was calculated as the band density value.

All data were analyzed with SPSS 20.0 software (SPSS Inc., Chicgo, IL, USA), and the statistical significance of differences between two groups was evaluated by Student's *t*-test; one-way ANOVA was used for more than two groups. The difference was considered to be significant at *p* <0.01(difference between different capital letters is extremely significant) and *p* <0.05(differences between different lowercase letters); the data was expressed as mean ± SD.

## Results

### Development of pcwaaF and rwaaF

#### Cloning the waaF Gene

**Figure 1:**
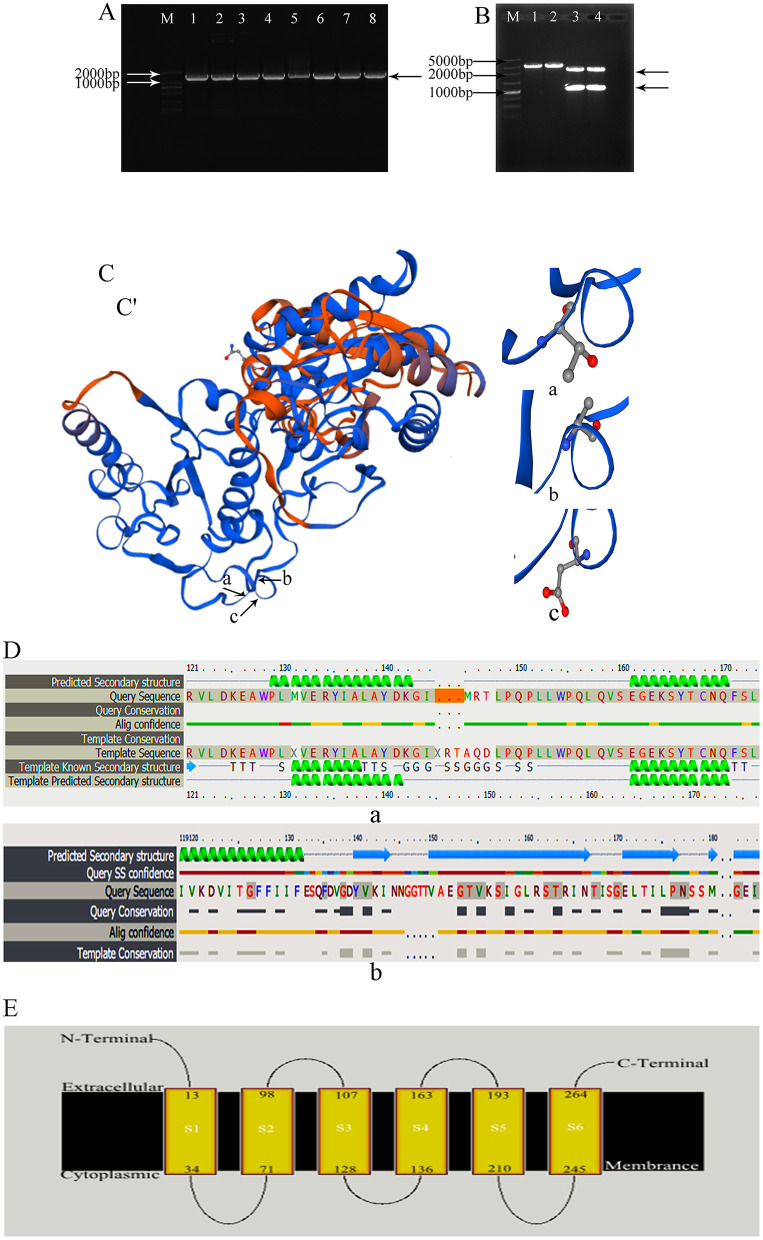
**(A)** waaF gene amplification by PCR; M: 2,000 bp Marker. 1–8: amplified the waaF gene band which appeared near the 1,000 marker's band. **(B)** Plasmid vector PGEM-T-waaF enzyme digestion; M: 5,000 bp Marker. 1, 2: pGEM-T-waaF plasmid; 3, 4: pGEM-T-waaF plasmid was digested by *BamH* and *Kpn* I, the waaF genes was near 1,000 marker's band, and the other was pGEM. **(C,C')** the three-dimensional structure of the mutant waaF. (a–c) Are sites of amino acid which are corresponding gene deletion; (a) the missing amino acid Alanine corresponding to ACA genes; (b) the missing amino acid Glutamine corresponding to AAG genes; (c) the missing amino acid Aspartic corresponding to GCA genes. **(D)** The amplified sequence translation protein was analyzed by Phyre2. (a) The lack of 142–150 genes are expressed the Alanine (A), Glutamine (Q), and Aspartic acid (D), (the orange band); (b) template sequence; Thicker gray lines indicate high conservatism, thinner gray lines indicate moderate conservatism. Correspondingly, in Alig confidence, the red line is confidence. **(E)** The A, Q, and D in S4 did not participate in extracellular membrane activities directly.

In previous research, we had isolated the wild-type *E. coli* from mastitis milk and extracted total DNA to use as templates. The 1,046 bp waaF gene was successfully amplified (Figure 1A) and inserted into pGEM-T-Easy vector and a new recombinant pGEM-T-waaF constructed. After that, it was digested with *BamHI* and *Kpn*I (Figure 1B) and a specific band found near 1,000 bp. Sequencing showed that the target fragment was 1,036 bp. Compared with the NCBI database (basic local alignment search tool), the waaF gene that had been mutated was digested in pGEM-T-waaF, and the homology was 99.7% with the reported sequence of *E. coli*. The result of comparison showed the 442–450, 1,046, and 10 genes were deleted, and no other point metamorphosis or frameshift was found. In order to analyze the effect of mutations on transcripts and evaluate the biological activity of cloned gene sequences, we predictively translated the sequencing genes and constructed the protein by the SWISS-MODEL, which was used to compare the difference of the three-dimensional structure and the two-dimensional structure of the mutations. The three-dimensional structure of the mutant waaF is shown in Figures 1C,C'. Its folding quantity, angle, stretching trend, and other structures are the same as that of the template. As shown in Figure 1C (a, b, and c), they were sites of amino acids with the corresponding gene deletion, ACAAAGGCA. After that, we analyzed the secondary structure of the waaF mutation. Figure 1D (a) shows the lack of 142–150 expressing the alanine (A), glutamine (Q), and aspartic acid (D), whereas, compared with the template sequence shown in Figure 1D (b), there is no conserved sequence (In Query Conservation, thicker gray lines indicate high conservatism, while thinner gray lines indicate moderate conservatism. Correspondingly, the red line is confidence). As shown in Figure 1E, the A, Q, and D in S4 does not participate in extracellular membrane activities directly, so, they were meaningless mutations.

#### Construction of pcwaaF

**Figure 2:**
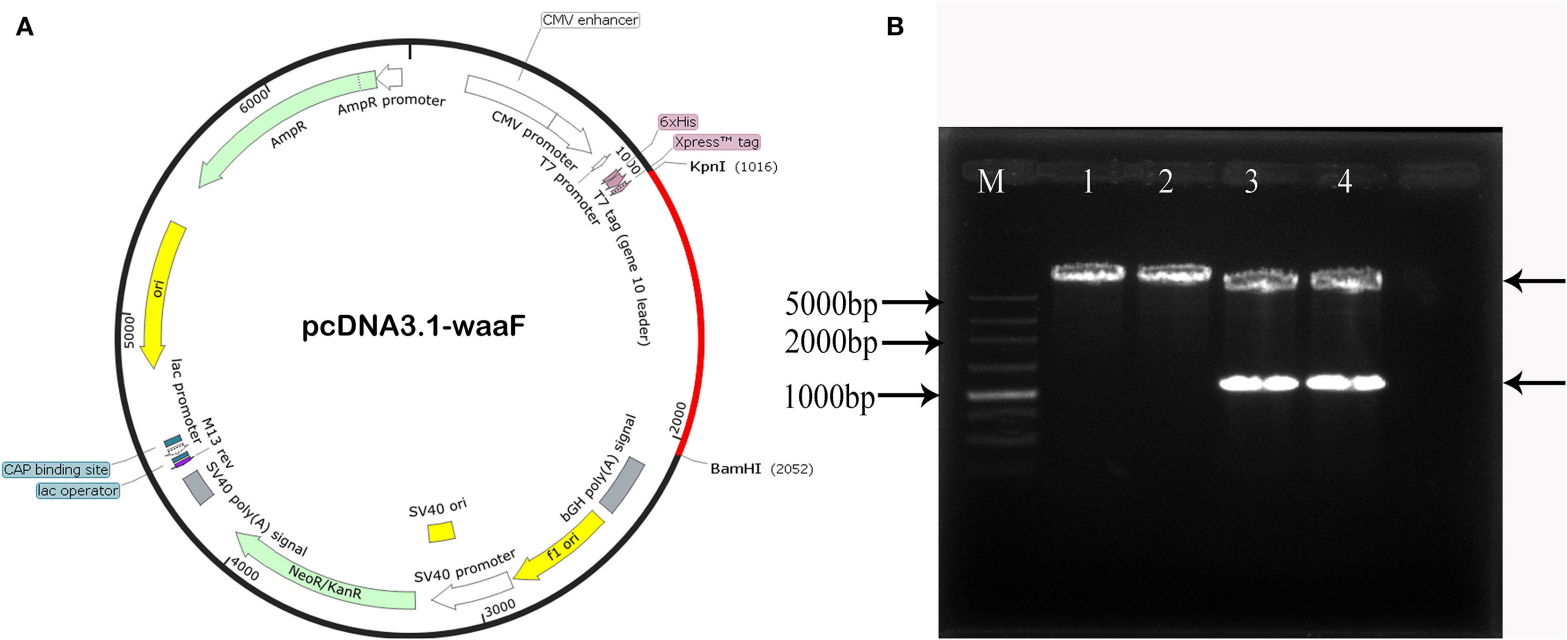
**(A)** The new recombinant eukaryotic pcwaaF plasmid. The red part is the sequence of waaF genes inserted into the vector. **(B)** *BamHI* and *Kpn*I were digested the pcwaaF. Lane 1, 2 pcDNA 3.1-*waaF* plasmid; Lanes 3, 4 pcDNA 3. 1-waaF plasmid digested with *BamH* and *Kpn* I, and the band near 1,000 marker was waaF gene and the above band was pcDNA 3.1 plasmid; Lane M, 5,000 markers.

The new constructed recombinant eukaryotic pcDNA3.1-waaF plasmid is shown in Figure 2A. The red part is the sequence of waaF genes inserted into the vector. The *BamHI* and *Kpn*I were digested with pcDNA3.1-waaF to 5,000 and 1,000 bp (Figure 2B). The results of sequence analysis showed that the pcwaaF was successfully constructed and OD (A260/280) was 1.867.

#### Construction of rwaaF

##### Transfection and Expression

**Figure 3:**
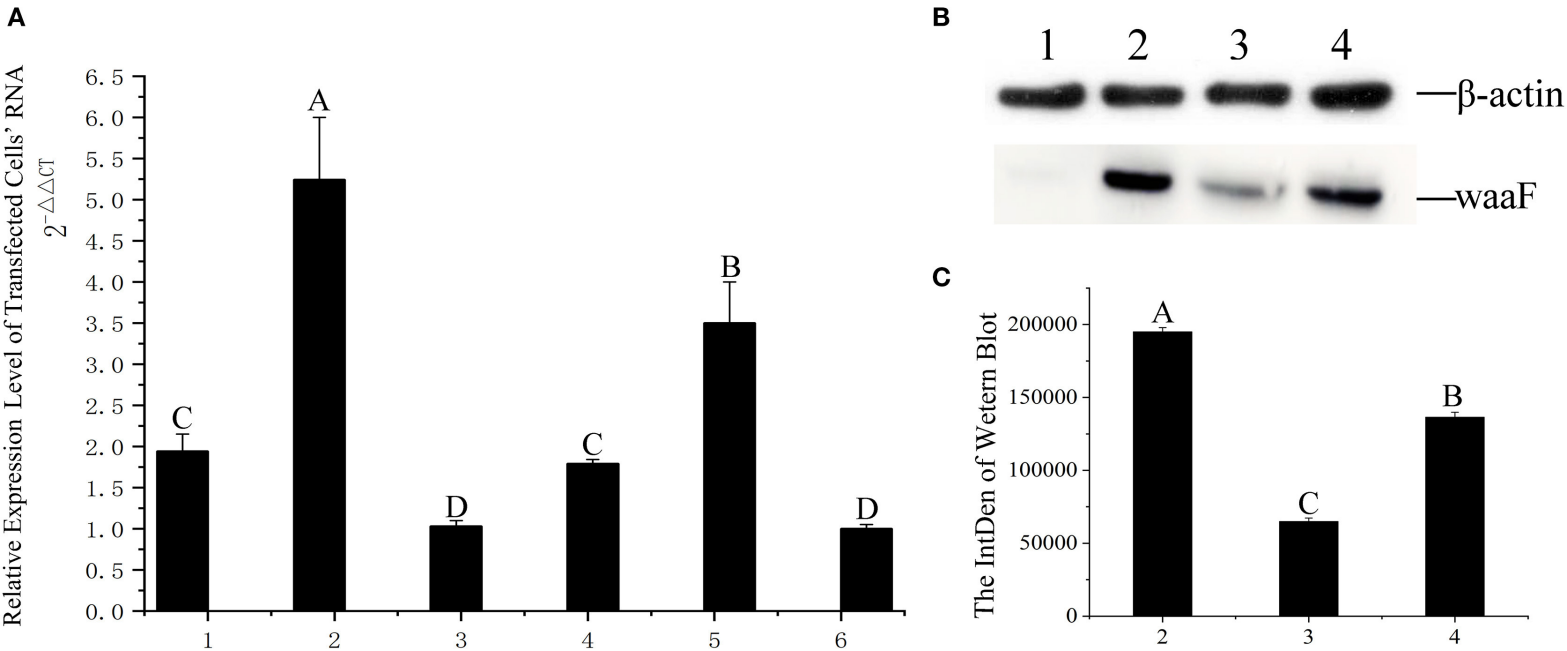
**(A)** qRT-PCR was applied for assaying the level of expression at RNA level, 1–6: The same positive cells were cultured in six vials, their RNA extracted, and numbered 1–6, respectively; the difference between different capital letters is extremely significant (*p* <0.01). **(B)** The expression of waaF protein. Lane 1, protein of MCF-7 cells. Lane 2–4, the waaF expression was observed in No. 2, No. 3, and No. 4 cells. **(C)** The curve of the western blot expression band; the difference between different capital letters is extremely significant (*p* <0.01).

With a screening concentration of G418 at 800 μg/ml, the mRNA expression of transfected cells was determined by qRT-PCR. As shown in Figure 3A, the expression of the No.2 cell is significantly higher than others (*p* <0.01), and the Cq of all qPCR data were <30; the melting curve had one only one specific peak.

We chose the No.3 and No.4 cell as control with No.2 in western blotting.

Western blotting verified that the waaF expression protein of the No.2 cell was significantly higher than that of No.3 and No.4 (*p* <0.01), as shown in Figures 3B and C. The molecular weight of waaF was ~40 kDa, very similar to the expected size of 36.67 kDa.

The purification rate was about 91.3% by HPLC C18 column, and the rwaaF was successfully constructed.

### Safety Assessments

**Figure 4:**
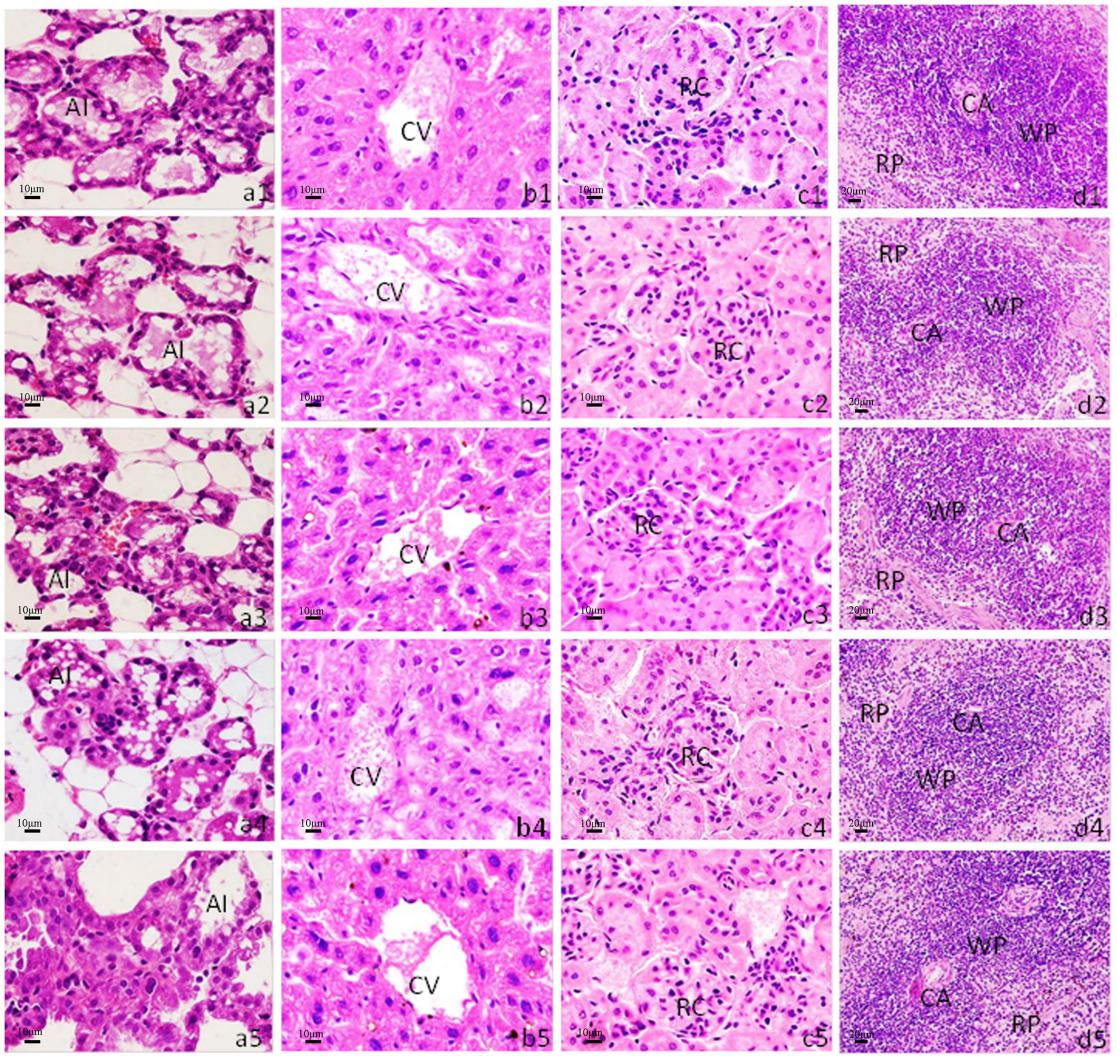
Histological analysis of mouse mammary gland, liver, kidney, and spleen. H&E staining. The scale bar indicated as 10 μm for mammary gland, liver, kidney, and 20 μm for spleen. **(a1–a5)** Histological sections of mice mammary gland; **(b1–b5)** liver; **(c1–c5)** kidney; **(d1–d5)** spleen. **(a1–d1)** The tissues from control group which were inoculated the 80 μg PBS; **(a2–d2)** inoculated the 60 μg rwaaF; **(a3–d3)** inoculated the 80 μg rwaaF; **(a4–d4)** inoculated the 60 μg pcwaaF; **(a5–d5)** inoculated the 80 μg pcwaaF. AI, acinus; CA, central artery; CV, central vein; RC, renal corpuscle; RP, red pulp; WP, white pulp.

We administered the amount of 3- and 4-times dosage of pcwaaF/rwaaF injection to mice and evaluated the safety level. Within 1 h after injection, the mice immunized with 60 and 80 μg pcwaaF showed torpor, especially those injected with 80 μg. After 1.5 h, the symptoms of the mice improved, and 2 h later, they ate and drank water normally. Furthermore, as shown in Tables 4, 5, there were no significant differences between all the vaccine groups and the control group in complete blood count and blood biochemical profiles (*p* > 0.05). Beyond that, we observed the mammary glands, liver, kidney, and spleen of pathological sections in each group. The results are shown in Figure 4; there were no obvious pathological changes observed in each tissue. Under the microscope, the lactating breast epithelial cell was rich in lipid droplets and obvious secretions in the glandular cavity of the control group (Figure 4a1); in comparison, there were no obvious pathological damages in all vaccine groups (Figures 4a2–a5), but the breast glandular secretory structure was not obvious in 80 μg pcwaaF group. In the liver sections, the hepatic cord was clearly distributed surrounding the central vein (Figure 4b1). The hepatic cords in the 60 μg rwaaF and pcwaaF groups (Figures 4b2,b4) did not have obvious pathological changes, whereas the hepatic cords were slightly swollen in the 80 μg rwaaF and pcwaaF group (Figures 4b3,b5). In the kidney sections, the glomerular structure of the control group was clear (Figure 4c1), and there were no obvious pathological changes in the other vaccine groups. In the spleen sections, the white and red pulp around the central artery of the spleen was distinct (Figure 4d1); the central artery was clear in the 60 and 80 μg rwaaF groups. Meanwhile, there was an obvious boundary between white pulp and red pulp (Figures 4d2,d3), but in the 60 and 80 μg pcwaaF groups, the number of white pulp lymphocytes were significantly decreased, the center artery was swollen, and there was no significant change in the red pulp (Figures 4d4,d5).

**Table 4:** The results of hematologic about vaccinated BALB/c mice which were vaccinated rwaaF (60 and 80 μg), pcwaaF (60 and 80 μg), and PBS, respectively.

**Group**	**PBS**	**rwaaF**	**rwaaF**	**pcwaaF**	**pcwaaF**	***p-*value**
		**60 μg**	**80 μg**	**60 μg**	**80 μg**	
RBC (×10^12^·L^−1^)	10.35 ± 0.36	10.65 ± 0.37	10.61 ± 0.49	10.64 ± 0.32	10.18 ± 0.24	>0.05
HGB (× g·dL^−1^)	14.27 ± 3.64	14.41 ± 2.68	14.5 ± 2.93	14.0 ± 2.83	14.0 ± 3.72	>0.05
WBC (×10^9^·L^−1^)	8.3 ± 2.54	8.52 ± 2.15	8.50 ± 2.40	8.74 ± 3.09	8.48 ± 1.96	>0.05
HCT (%)	44.13 ± 7.76	42.98 ± 7.35	43.48 ± 7.13	40.38 ± 13.9	43.44 ± 9.46	>0.05
MCH (pg)	13.51 ± 2.03	13.25 ± 2.71	13.43 ± 2.14	13.79 ± 2.44	13.14 ± 2.35	>0.05
MCHC (g·L^−1^)	33.6 ± 2.06	33.27 ± 2.83	31.13 ± 6.80	33.35 ± 2.85	31.99 ± 3.88	>0.05
RDW (CV/fL)	18.50 ± 4.09	18.23 ± 2.85	18.22 ± 1.47	18.18 ± 1.82	18.36 ± 2.25	>0.05
PLT (×103/μl)	748.5 ± 176.23	757.31 ± 113.60	749.87 ± 119.64	751.03 ± 61.18	755.25 ± 175.52	>0.05

**Table 5:** The results of Serum biochemical in vaccinated BALB/c mice which were vaccinated rwaaF (60 and 80 μg), pcwaaF (60 and 80 μg), and PBS, respectively.

**Group**	**PBS**	**rwaaF**	**rwaaF**	**pcwaaF**	**pcwaaF**	***p-*value**
		**60 μg**	**80 μg**	**60 μg**	**80 μg**	
ALT (U·L^−1^)	32.12 ± 12.52	32.50 ± 7.69	31.98 ± 7.98	32.56 ± 7.92	35.08 ± 11.93	>0.05
AST (U·L^−1^)	111.42 ± 22.40	111.2 ± 19.93	111.40 ± 21.56	113.79 ± 19.38	112.26 ± 16.21	>0.05
ALP (U·L^−1^)	84.99 ± 9.77	86.23 ± 13.98	85.23 ± 11.79	82.89 ± 13.83	81.75 ± 10.73	>0.05
CR (μmol·L^−1^)	84.43 ± 14.04	82.29 ± 19.42	84.36 ± 16.18	85.80 ± 17.37	84.14 ± 13.99	>0.05
ALB (g·L^−1^)	30.21 ± 5.21	30.36 ± 5.10	29.64 ± 5.18	31.34 ± 5.30	32.06 ± 5.46	>0.05
GLU (mmol·L^−1^)	4.32 ± 1.66	4.03 ± 0.45	3.46 ± 0.59	3.12 ± 0.87	3.20 ± 1.43	>0.05
BUN (mmol·L^−1^)	9.39 ± 2.55	9.05 ± 1.51	9.15 ± 0.98	9.22 ± 1.68	6.20 ± 2.30	>0.05
TBIL (μmol·L^−1^)	1.92 ± 0.87	1.93 ± 1.53	1.96 ± 0.78	2.02 ± 1.26	1.9 ± 0.95	> 0.05
TP (g·L^−1^)	71.12 ± 9.35	72.10 ± 6.97	72.02 ± 7.24	72.38 ± 6.69	73.02 ± 5.56	>0.05

*(n = 10). RBC, total number of red blood cells; HGB, hemoglobin; WBC, white blood cells; HCT, hematocrit; MCH, mean corpusular hemoglobin; MCHC, mean corpusular hemoglobin concentration; RDW, red blood cell distribution width; PLT, platelet count; ALT, alanine aminotransferase; AST, aspartate aminotransferase; ALP, alkaline phosphatase; CR, creatinine; ALB, albumin; GLU, glucose; BUN, blood urea nitrogen; TBIL, total bilirubin; TP, total protein*.

**Figure 5:**
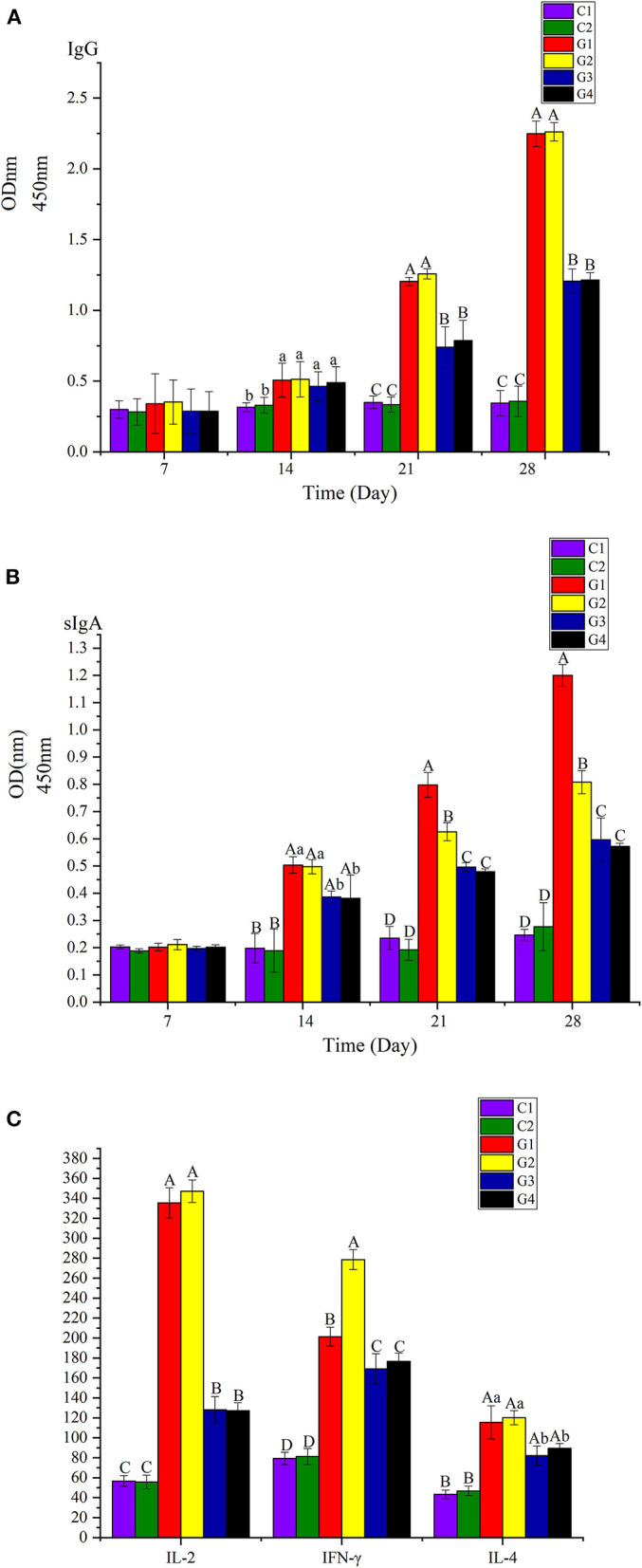
**(A)** Analyzed the IgG on the 7th, 14th, 21th, and 28th days. **(B)** Analyzed the sIgA of feces on the 7th, 14th, 21th, and 28th days. **(C)** Analyzed the IL-2, IL-4, and IFN-γ of serum on the 28th day. The difference between different capital letters is extremely significant (*p* <0.01), and the significant differences between different lowercase letters (*p* <0.05).

### Immunogenicity—IgG, sIgA, IL-2, IL-4, and IFN-γ Were Detected by ELISA

We analyzed IgG in serum and sIgA in feces the 7th, 14th, 21st, and 28th days atter injection. We also analyzed IL-2, IL-4, and IFN-γ in serum on the 28th day. Among the groups, the levels of IgG, sIgA, IL-2, and IFN-γ in G1 and G2 were significantly higher than in G3 and G4 (*p* <0.01); the levels of IL-4 in G1 and G2 were higher than in G3 and G4 (*p* <0.05). There were no differences in IgG, IL-2, and IL-4 between G1 and G2 (*p* > 0.05); however, the level of sIgA in G1 was significantly higher than in G2 (*p* <0.01), but the level of IL-4 in G2 was significantly higher than in G1 (*p* <0.01).

### Challenge Test

**Figure 6:**
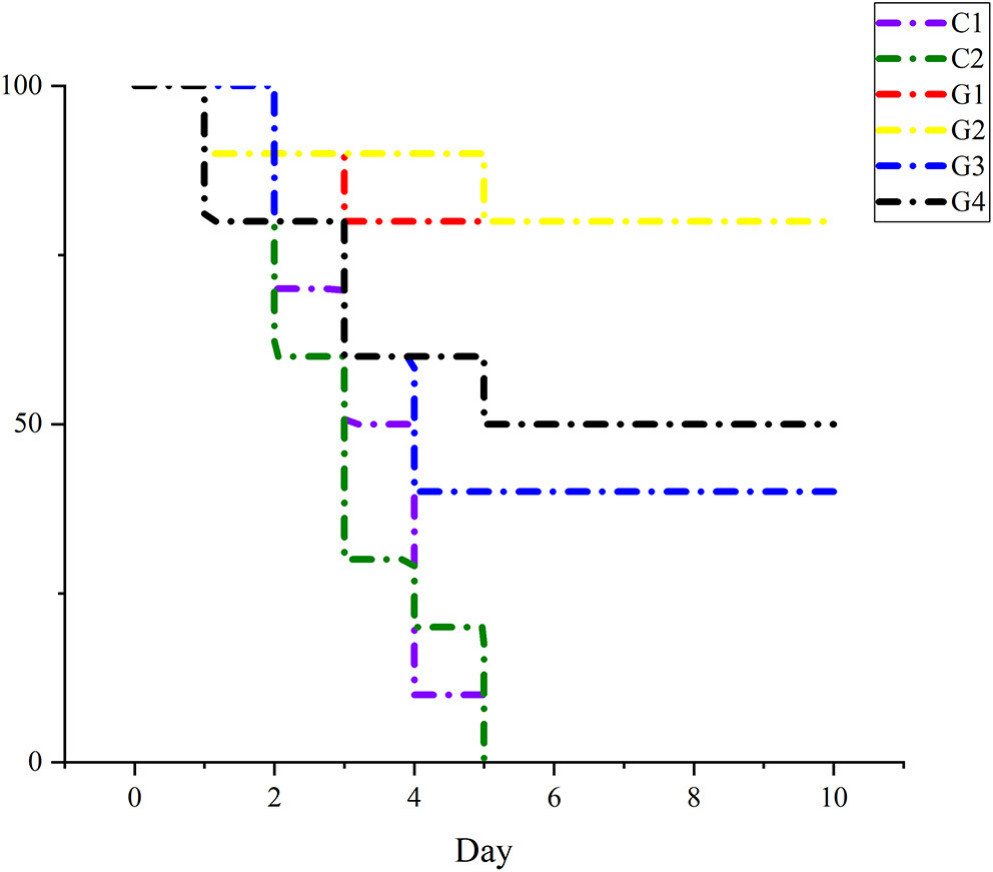
The percent of survival rate of mice after being challenged with *E. coli* to each group.

After being injected 4 times with an *E. coli* dose of 4 × 10^6^ CFU, the mice in G1 and G2 were good mentally and emaciation was not obvious. Among them, a mouse died on the 2nd and 4th days in G1 and a mouse died on the 2nd and 6th days in G2; the overall survival rate was 80%, whereas the weight loss of mice in G3 and G4 was more obvious than that in G1 and G2. At the same time, the fur was rough, they were depressed, and the feed intake was less; after the 3rd day, they recovered gradually. In G3, two mice each died on the 3rd, 4th, and 5th day, and the survival rate was 40%. Meanwhile, in G4, two died on the 2nd and 4th days, and 1 died on the 6th day, and the survival rate was 50%. Compared with vaccine groups, the weight loss, rough fur, lack of eating, and somnolence of the mice in the C1 and C2 were more obvious. In C1, three died on the 2nd day, two on the 3rd day, four on the 4th day, and the reminder on the 5th day, while in C2, four died on the 2nd day, three on the 3rd day, one on the 4th day, and the reminder died on the 5th day. Details are in Figure 6.

## Discussion

In a previous study, the research team collected 380 milk samples from dairy cows with clinical mastitis from dairy farms in Gansu, Ningxia, and Qinghai. The detection rate of *E. coli* was as high as 83.9% (21); therefore, a more cost-effective vaccine needs be developed to prevent *E. coli* mastitis in cows (22, 23). LPS is the key pathogenic factor in *E. coli* (24), and waaF is the core component with a highly conserved O-antigen gene in LPS that catalyzes the transfer of the second L-glycero-D-manno-heptose residue to the core oligosaccharide moiety of LPS (25). It not only affects the colonization, adhesion, and invasiveness of the pathogen (14), but it is also related to pro-inflammatory factors in the host (15). In addition, the gene of waaF is highly specific and highly consistent with the phenotype of other Gram-negative bacteria (25–28). Oldfield (25) and Chandan (29) added the waaF gene of *campylobacter* and *Helicobacter pylori* to the mutant *Salmonella typhimurium* lacking waaF, respectively, which not only restored the structure of core oligosaccharides, but also improved the adhesion and invasion of mutants. In this study, waaF was selected as the amplifying gene to construct the subunit and DNA vaccines for fundamentally inhibiting the binding of LPS to the target site. In addition, the immunogenicity and potential of two different forms of vaccines were compared and analyzed.

The eukaryotic vector is the main body of the DNA vaccine. The stronger the ability of the vector to express the antigen protein, the stronger the immune response induced in the host is (30). Some studies indicated that the waaF gene of *Bordetella pertussis* expressed in pBluescriptII (31) and *Vibrio parahaemolyticus* waaF cloned into pBBR1MCS2 (32) have prokaryotic expression. At present, there is no report on the construction of a eukaryotic vector of the waaF gene. In this study, the target gene was first cloned into pEGM-T, cut by *KpnI* and *BamH*, linearized by pcDNA3.1, and then the subcloned gene was expressed in the eukaryotic vector pcDNA3.1 and the pcwaaF was constructed. The successful development of pcwaaF was verified by sequencing.

The waaF gene sequence was transferred into MCF-7 cells in this study. There are many methods of transfection, and electroporation and liposome transfection are routinely used. Among them, electroporation has the highest transfection efficiency, which can reach more than 90% (33), but it can interfere with the key biological properties of cells, such as proliferation, metabolism, and gene expression (34). If the conditions of liposome transfection are optimized, the effect of liposome transfection is better than that of electroporation (35). Therefore, we selected G418 for transfection and concluded that 800 μg/mL was the lowest lethal dose. The WB results showed that the protein size was about 36 KDa, which was like the predicted results, and the rwaaF was successfully constructed.

The subunit vaccine is one of the most promising vaccines at present (36). Subunit vaccines for of hepatitis B, influenza, and pertussis (37) have been licensed for human use. Studies have been reported that the EspA subunit vaccine may become the first marketed vaccine against *E. coli* O157:H7 (38).

In vaccine research, low production cost, low price, and easy storage are key factors; however, safety is an important index for the utilization and promotion of the vaccine (39). At present, the universal vaccine for the prevention and treatment of coliform cow mastitis is the J5 vaccine which is inactivated *E. coli* vaccine with an incomplete O-antigen (39), and there are still some safety problems. It was found that the intraperitoneal injection of J5 inactivated vaccine could cause obvious toxic reactions in mice, such as rough coat, depression, hepatocyte enlargement, and inflammatory infiltration (39). Rainard reported that the J5 immune serum was not an improvement on the already high efficiency of naturally acquired antibodies to *E. coli* (40). However, the safety and effectiveness of the J5 vaccine in dairy cows has not been reported clearly (41). In this research, BALB/c mice were used to evaluate the safety at three to four times the immunization dose. The experimental mice did not have a poor mental state, and their hematological and blood biochemical indicators were within the normal range; beyond that, the mammary glands, liver, kidneys, and spleen of the mice did not show obvious pathological changes. This demonstrates that the DNA and subunit vaccines are safer than the J5 vaccine. The mice injected with the pcwaaF showed less energy and moved slowly, and the performance effects of a high dose were more obvious. The damage to mice in the rwaaF group was less severe than in the pcwaaF group. Therefore, rwaaF is safer than pcwaaF. The results of this study provide a very valuable basis for the promotion and application of the vaccine.

To compare the immunogenicity of rwaaF and pcwaaF, this study analyzed the IgG in serum and sIgA of feces on the 7th, 14th, 21st, and 28th days after injection, and detected IL-2, IL-4, and IFN-γ in serum on the 28th day. The results of ELISA showed that all the factors in the vaccine groups were significantly higher than in the control groups, and the levels of IgG in the 20 and 40 μg rwaaF groups were significantly higher than in the 20 and 40 μg pcwaaF groups. Beyond that, the effect of humoral immunity had nothing to do with the dose. This is consistent with comparison of the rwaaF and pcwaaF of *Corynebacterium pseudotuberculosis* acid phosphatase CP01850 (42); sIgA exists in the secretions of the nasal cavity, bronchus, and gastrointestinal juice, and it is the main antibody produced by mucosal immunity and an important indicator of mucosal immunity (43). The vaccine activated humoral immunity as well as mucosal immunity (44). The detection results of sIgA in feces were similar to those of IgG. However, the sIgA of the 20 μg rwaaF group was significantly higher than that of the 40 μg rwaaF group, further indicating that the effect of mucosal immunity is also dose independent. After immunization of mice with rwaaF/pcwaaF, because of the effect of TI and T2 (45), the levels of IL-2, IFN-γ, and IL-4 all increased and stimulated T and B lymphocytes to differentiate in different directions (46). Although both the rwaaF and pcwaaF stimulated cellular immunity, the detection of IL-2, IFN-γ, and IL-4 has further confirmed that the rwaaF is more effective than the pcwaaF. Among all the factors tested, only the 40 μg rwaaF group was IFN-γ-extremely significantly higher than in the 20 μg rwaaF group. In the challenge test, the survival rates of the 20 and 40 μg rwaaF groups were both 80%, and the survival rates of the 20 and 40 μg pcwaaF groups were 40 and 50%, respectively, while all the mice in the control group died. This shows that both rwaaF and pcwaaF produced immune protection in mice, and the rwaaF gave better protection. In summary, rwaaF is more immunogenic and has a better inoculation effect than pcwaaF; the analysis of this study proves that 20 μg rwaaF is more economical and practical than 40 μg, which provides a reference for subsequent clinical applications.

However, while waaF DNA vaccine and subunit vaccine could induce the humoral and cellular immunity in mice and show ideal immune protection effects, they have not been evaluated in dairy cows. In addition, DNA vaccine may be integrated with the chromosomal genome of the host cells (47–50), leading to cell transformation, canceration, etc. Further, the complex pathogenesis of bovine mastitis is closely related with pathogens, host immunity, internal environment, and other factors. So, there is still a gap in the research regarding enhancing immunity between waaF DNA vaccine and subunit vaccine, adapting to the internal environment of dairy cows, and synergizing host immunity. In conclusion, waaF DNA vaccine and subunit vaccine will be candidates for *E. coli* mastitis vaccine, and bring a new opportunity for prevention and treatment of *E. coli* mastitis.

## Conclusion

This study demonstrates that waaF is a potential virulence factor in *E. coli*, and induced different immune responses as a purified recombinant subunit vaccine and DNA vaccine. The immune response elicited by waaF as a subunit vaccine is much stronger than DNA vaccine in murine model. These results suggest the utility of a new *E. coli* vaccine and provide a rationale for further investigation of bovine mastitis therapy and management.

## Data Availability Statement

The datasets presented in this study can be found in online repositories. The names of the repository/repositories and accession number(s) can be found in the article/supplementary material.

## Ethics Statement

The animal study was reviewed and approved by the Academic Committee of Gansu Agricultural University.

## Author Contributions

HW put forward the research concept and design. TW, LC, and FL performed the data analysis and drafting the article. JS and YZ carried out the experiment and provided part of the test results for the manuscript. HW edited the manuscript for approval for submission. LY agreed to be accountable for all aspects of the work in ensuring that questions related to the accuracy or integrity of any part of the work are appropriately investigated and resolved. All the authors have approved the final version of the manuscript.

## Funding

This work was supported by the fund of Gansu Key Laboratory of Animal Generational Physiology and Reproductive Regulation [Grant No. 20JR10RA563]; College of Veterinary Medicine, Gansu Agricultural University [Grant No. GAU XKJS 2018 056] and the fund of Innovation Star project of excellent postgraduates in Gansu Province [Grant No. 2021CXZX 358].

## Conflict of Interest

The authors declare that the research was conducted in the absence of any commercial or financial relationships that could be construed as a potential conflict of interest.

## Publisher's Note

All claims expressed in this article are solely those of the authors and do not necessarily represent those of their affiliated organizations, or those of the publisher, the editors and the reviewers. Any product that may be evaluated in this article, or claim that may be made by its manufacturer, is not guaranteed or endorsed by the publisher.
